# The selective MEK1 inhibitor Selumetinib enhances the antitumor activity of everolimus against renal cell carcinoma *in vitro* and *in vivo*

**DOI:** 10.18632/oncotarget.15346

**Published:** 2017-02-14

**Authors:** Yun Zou, Jianfeng Wang, Xuejiao Leng, Jiwei Huang, Wei Xue, Jin Zhang, Yiran Huang

**Affiliations:** ^1^ Department of Urology, Renji Hospital, School of Medicine, Shanghai Jiao Tong University, Shanghai, China; ^2^ Department of Oncology, Shanghai Chest Hospital, Shanghai Jiao Tong University, Shanghai, China

**Keywords:** renal cell carcinoma, everolimus, selumetinib, targeted therapy, combination therapy

## Abstract

Renal cell carcinoma (RCC) is a urologic malignant cancer and often diagnosed at an advanced stage, which results in high mortality. Targeted therapy may improve the quality of life and survival of patients who are not suitable for nephrectomy. Everolimus, an mTOR inhibitor, is currently used as sequential or second-line therapy for RCC refractory to Sunitinib or sorafenib. However, its efficiency is palliative. In this study, we evaluated whether the antitumor activity of everolimus against RCC is enhanced by selumetinib, a selective MEK1 inhibitor. We discovered that everolimus in combination with selumetinib synergistically inhibited the proliferation of Caki-1, 786-O and 769-P cells *in vitro*. Mechanistically, this combination decreased p-RPS6 and p-4E-BP1 dramatically, which causes G1 cell cycle arrest and prevents reactivation of AKT and ERK. *In vivo*, the antitumor efficacy and pharmacodynamic biomarkers of the combination therapy were recapitulated in Caki-1 xenograft model. In addition, this combination treatment potently inhibited angiogenesis in xenograft models by impairing VEGF secretion from tumor cells. Our findings provide a sound evidence that combination of everolimus and selumetinib is a potential dual-targeted strategy for renal cell carcinoma.

## INTRODUCTION

Renal cell carcinoma (RCC) is the most common form of kidney cancer, with an estimated 338,000 new cases diagnosed and 144,000 deaths occurring worldwide every year [1]. Surgical resection is the only potentially curative therapy for this disease; however, for the approximately 30% of patients with metastatic disease at the time of diagnosis [2], nephrectomy is not a viable option. Therefore, systemic treatment may be provided since it can improve the quality of life and survival of patients [3]. Everolimus (RAD001), an oral mTOR inhibitor, was approved by the US Food and Drug Administration (FDA) and European Medicines Agency (EMEA) as a sequential or second-line therapy for advanced RCC refractory to Sunitinib or sorafenib [4]. However, in a phase 3 trial assessing patients with RCC refractory to EGFR-TKIs, everolimus was shown to slightly improve progression-free survival compared with placebo (median, 4.9 vs. 1.9 months) [5]. Hence, it is urgent to identify an optimal therapy to overcome this limited clinical benefit. Compared to single agents, combinatorial therapy seems to be potentially more successful in controlling cell signaling [6]. Motzer and colleagues demonstrated a synergistic effect of everolimus and Lenvatinib in patients with advanced or metastatic RCC, and this was the first successful combination therapy approved by FDA [7, 8]. So the well-known molecular mechanisms deserve potential probing and combination therapy is promising.

PI3K/AKT/mTOR and Ras/MEK/ERK are the most critical cell signaling pathways in carcinogenesis and disease progression, and cross-talks between these two pathways have been demonstrated [9–11]. Selumetinib (AZD6244) is an oral, non-ATP competitive, and highly selective MEK1 inhibitor, which is now in Phase 3 clinical trials for treating different types of solid tumors. Several studies have demonstrated that AZD6244 enhances the antitumor activity of everolimus in pediatric gliomas [12], neuroblastoma [13], and acute myelogenous leukemia (AML) [14]. However, only one study showed that treatment with sorafenib/AZD6244 combination enhances the antitumor activity of sorafenib in RCC [15]. Thus, in the present study, we evaluated the potential of a combined therapeutic approach with everolimus and selumetinib for the treatment of RCC, exploring the mechanisms of the resulting antitumor efficacy.

## RESULTS

### RAD001 and AZD6244 synergistically reduce the viability of RCC cells

**Figure 1:**
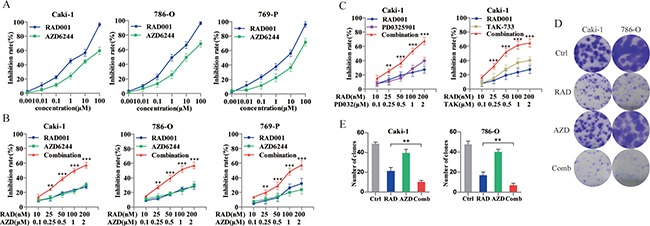
Identification of AZD6244 as a potential enhancer for combination therapy with RAD001 in RCC cells (**A**) Cells were treated with varying concentrations of RAD001 or AZD6244 alone for 72 hr. (**B**) Cells were treated with RAD001/AZD6244 on a fixed dose ratio 1:10 in combination for 72 hr. Calculated by the CompuSyn software according to the Chou-Talalay method, CI values at latter four paired concentrations were < 1. **p < 0.01; ***p < 0.001. (**C**) Caki-1 cells were treated with PD0325901 or TAK-733 (MEK inhibitors) in combination with RAD001 for 72 hr. CI values at latter four paired concentrations were < 1. **p < 0.01; ***p < 0.001. (**D**) Cells were seeded in 6-well plates at a density of 500 cells/well, exposing to 0.1 μM RAD001 (RAD), 1 μM AZD6244 (AZD), 0.1 μM RAD/1 μM AZD (Comb), or equivalent volume of DMSO (Ctrl). After 10 days of treatment, the colonies were stained with crystal violet and scanned. (**E**) Quantification of crystal violet staining from colonies in (D). ** *p* < 0.01.

Before evaluating the efficacy of RAD001 plus AZD6244 in human RCC cells, we first assessed their sensitivity to single compounds. After treatment with 0.001, 0.01, 0.1, 1, 10 and 100 μM of RAD001 or AZD6244 for 72 h, cell proliferation was analyzed by the SRB assay. As shown in Figure 1A, all cell lines were sensitive to RAD001 with IC50 values < 10 μM, while AZD6244 alone mildly suppressed the growth of cells, with slightly high IC50 values. Based on these data, a fixed dose ratio of 1:10 for RAD001 and AZD6244 was selected for the combination therapy. Cell proliferation at five paired concentrations were compared with monotherapy in Caki-1, 786-O and 769-P cells (Figure 1B). The synergistic effects of RAD001 and AZD6244 were reflected by the combination index (CI) calculated by the CompuSyn software according to the Chou-Talalay method [16]. CI values at latter four paired concentrations were < 1, suggesting that RAD001 and AZD6244 worked synergistically in inhibiting the growth of RCC cells. This result was recapitulated by combination of RAD001 with pan-MEK inhibitor PD0325901 or selective-MEK1 inhibitor TAK-733 in Caki-1 cells (Figure 1C). The inhibition rate reached approximately 50% when 0.1 μM RAD001 plus 1μM AZD6244 was used for treatment; therefore these amounts were used in subsequent experiments. The above results were further confirmed by the clonogenic assay in Caki-1 and 786-O cells (Figure 1D, 1E).

### Combination therapy in RCC cells enhances cell cycle arrest

**Figure 2:**
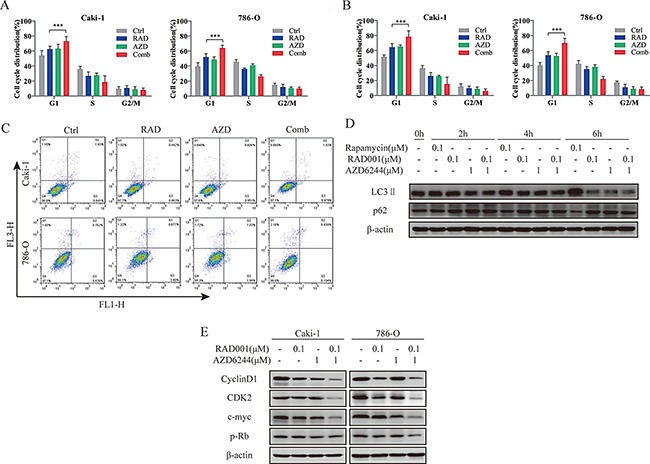
Induction of cell cycle arrest in RCC cells by combined treatment (**A**, **B**) Cells were treated with 0.1 μM RAD001 (RAD), 1 μM AZD6244 (AZD), 0.1 μM RAD/1 μM AZD (Comb), or equivalent volume of DMSO (Ctrl) for 24 hr (A) and 48 hr (B). Cell cycle distribution was determined by FACS analysis, and results are shown in the bar graph as percentages of G1, S and G2/M cells. An increased percentage of G1 phase was found for Comb group. ***p < 0.001. (**C**) Cells were treated with 0.1 μM RAD001 (RAD), 1 μM AZD6244 (AZD), 0.1 μM RAD/1 μM AZD (Comb), or equivalent volume of DMSO (Ctrl) for 48 hr. Apoptotic cells were detected by flow cytometric analysis (not significant). (**D**) Autophagy was detected by levels of LC3II/β-actin and p62 when Caki-1 cells were treated with indicated reagents (Rapamycin as positive control) for the indicated times. (**E**) Cell lysates were immunoblotted with antibodies of cell cycle regulation proteins after treatment with indicated inhibitors for 24 hr.

To further probe why combination of RAD001 and AZD6244 caused synergistic inhibition of cell growth, we investigated cell cycle distribution, apoptosis and autophagy on Caki-1 and 786-O cells. No significant differences of apoptosis and autophagy were observed (Figure 2C, 2D). However, significantly more cells were accumulated in the G1 phase after treatment with both agents compared with the monotherapy (Figure 2A, 2B). Moreover, Western blot demonstrated that treatment with the combination overtly reduced the expression levels of cyclin D1, CDK2, c-Myc and p-Rb in both Caki-1 and 786-O cells (Figure 2E); the latter proteins are involved in G1 to S transition. Thus, combination of AZD6244 inhibited cell proliferation by increasing RAD001-induced G1 cell cycle arrest.

### Effect of RAD001 and AZD6244 on signal transduction pathways in RCC cells

**Figure 3:**
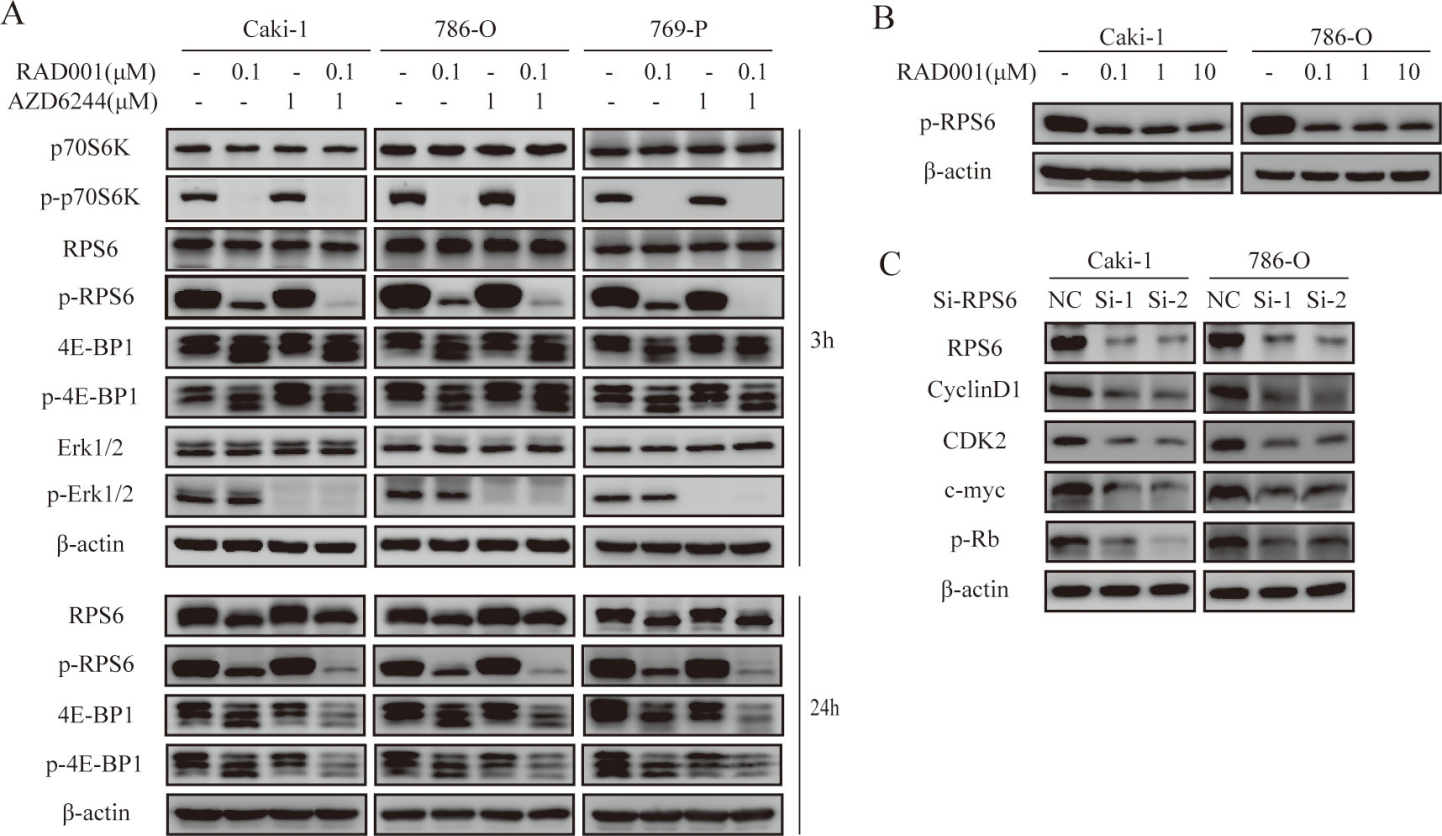
Effects of combination therapy on signaling pathways and RPS6 on cell cycle regulation (**A**) Cells were treated with indicated inhibitors for 3 hr and 24 hr. Western blot analysis performed with the cell lysates for the downstream effectors. (**B**) Levels of p-RPS6 were detected by Western blot when cells were treated with varying concentrations of RAD001 for 3 hr. (**C**) After knockdown of RPS6 for 48 hr, cell lysates were analyzed by Western blot to verify the expression of cell cycle proteins induced by RAD001 and AZD6244.

To assess the crosstalk between mTOR and MEK pathways, Western blot analysis was used to test the expression of downstream molecules in RCC cells. Interestingly, p-RPS6 appeared to be completely inhibited by RAD001, when combined with AZD6244 (Figure 3A). To eliminate the impact of low concentration of RAD001, we tested the p-RPS6 levels at different concentration of RAD001 from 0.1 to 10 μM (Figure 3B). The results certified that RAD001 alone could not block the p-RPS6 levels and the addition of AZD6244 was necessary for the thorough blockage. Loss of t-RPS6 and p-RPS6 greatly suppresses NSCLC cell viability by inducing G1 cell cycle arrest, along with decreased CDK2, CDK4, cyclin D1, cyclin E1 and p-Rb levels [17]. Moreover, depletion of S6 results in a sharp decrease of cyclin D1 and CDK2 levels to regulate cell viability in esophageal squamous cell carcinoma [18]. Then we confirmed this in RCC using a sequence-specific siRNA targeting RPS6. As shown in Figure 3C, the expression levels of cyclin D1, CDK2, c-Myc and p-Rb were markedly reduced after RPS6 silencing. These results suggest that AZD6244 enhances the antitumor effect of RAD001 by strengthening p-RPS6 inhibition, which causes G1 cell cycle arrest in RCC. In addition, we discovered that combination of RAD001 and AZD6244 caused effective inhibition of 4E-BP1 and p-4E-BP1 synergistically after 24 hr treatment (Figure 3A). It was consistent with previous findings that combined inhibition of ERK and AKT effectively inhibits 4E-BP1 phosphorylation and prevents reactivation of ERK and AKT [19].

### Effect of RAD001 and AZD6244 on angiogenesis

**Figure 4:**
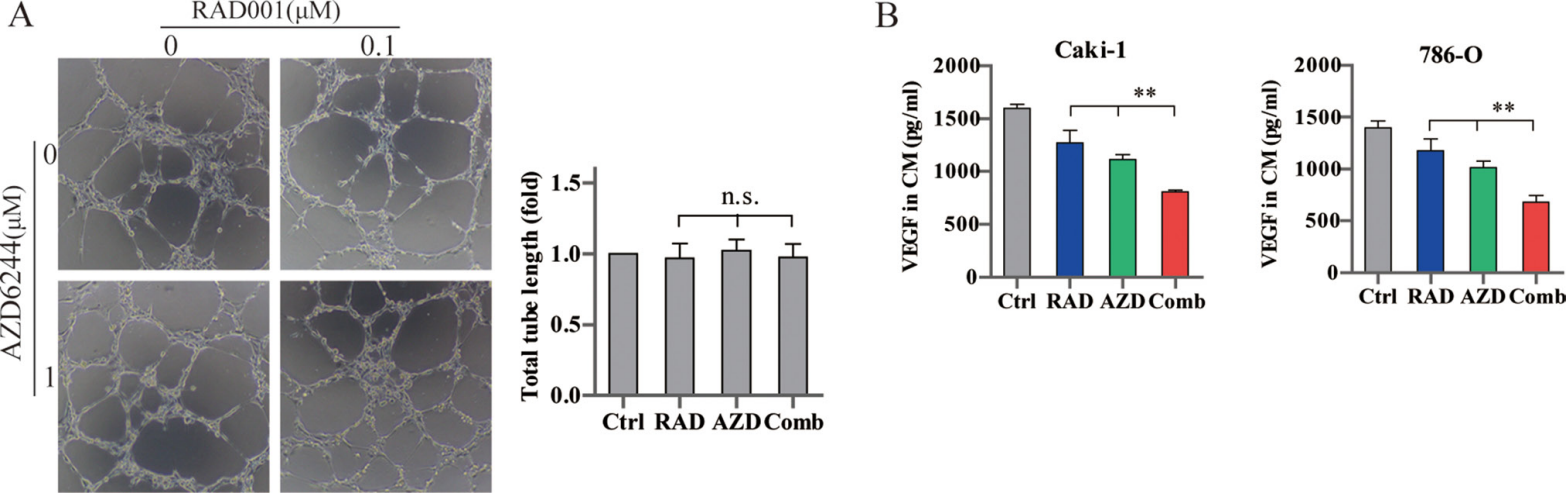
Effects of combined RAD001 and AZD6244 treatment on angiogenesis (**A**) HUVEC cells (10^4^ per well) with conditioned medium were seeded on top of Matrigel in 96-well plates for 4 hr. Tube formation was pictured with a bright field microscope. The tube lengths were quantified with the ImageJ software. n.s., not significant. (**B**) RCC cells were deprived of serum and treated with 0.1 μM RAD001 (RAD), 1 μM AZD6244 (AZD), 0.1 μM RAD/1 μM AZD (Comb), or equivalent volume of DMSO (Ctrl) for 24 hr. VEGF levels in the supernatants were assessed by ELISA. **p < 0.01.

**Figure 5:**
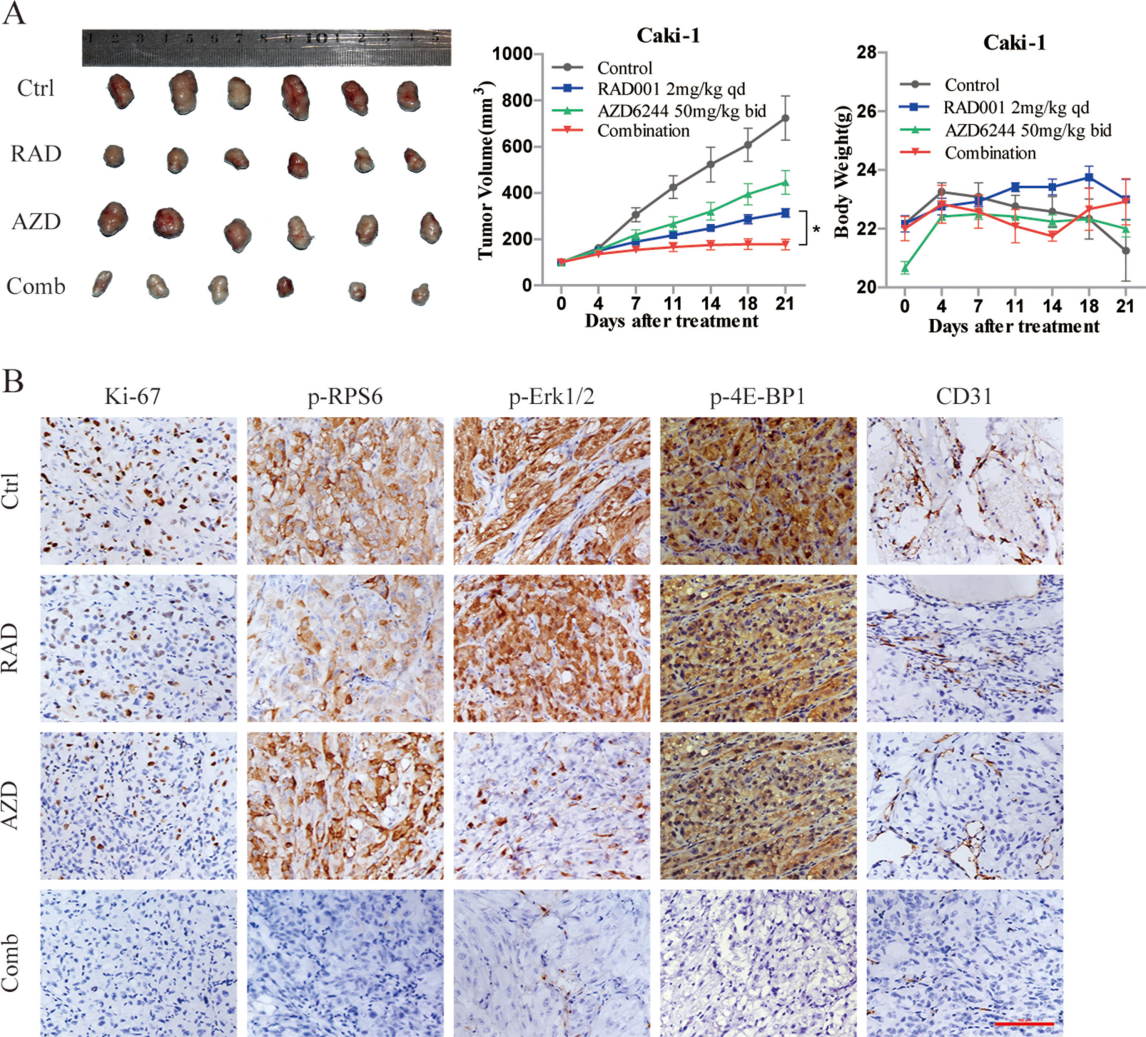
Efficacy of combined RAD001 and AZD6244 treatment *in vivo* (**A**) Caki-1 xenografts mice were treated with RAD001 (2 mg/kg daily), AZD6244 (50 mg/kg twice daily) or both drugs in combination for 21 days. Tumor tissues at treatment end were shown (left panel). Tumor volumes (middle panel) and body weights (right panel) of mice from 0 to 21 days were expressed as mean ± SEM, *n* = 6. *p < 0.05. (**B**) Tumor tissues from Caki-1 xenografts were resected and immunostained with Ki-67, p-RPS6, p-Erk1/2, p-4E-BP1 and CD31 antibodies. Magnification, ×200. Scale bar represents 100 μm.

To explore whether the combination therapy affected angiogenesis, we examined tube formation with human umbilical vein endothelial cells (HUVECs), and assessed VEGF levels secreted from RCC cells. Neither single drugs nor combination treatment suppressed HUVEC tube formation (Figure 4A). While VEGF secretion from RCC cells were markedly reduced after treatment with RAD001 and AZD6244 in combination (Figure 4B), which was consistent with the microvessel density in xenograft tumor stained with CD31 (Figure 5B). Our data suggest that these drugs inhibit angiogenesis by impairing VEGF secretion rather than as a direct effect on vascular endothelial cells.

### AZD6244 significantly enhances the antitumor efficacy of RAD001 in RCC xenograft tumors

For the establishment of subcutaneous tumor models, the metastatic Caki-1 cells were selected, since it yields better tumorigenesis. To verify whether combination therapy provides broad antitumor activity *in vivo*, RAD001 and AZD6244 were administered alone and in combination, respectively, to mice for 21 days. As shown in Figure 5A, treatment with RAD001 caused an approximately 43% reduction in tumor size, whereas AZD6244 treatment slightly inhibited tumor growth with T/C value of 61.6%. In contrast, tumor volumes in combination group were almost completely blunted after 21 days of treatment. In terms of tolerance and toxicity, no significant weight loss or death was observed in either group of mice. In agreement with the mechanistic findings *in vitro*, IHC analysis of tumor tissues demonstrated that combined treatment with RAD001 and AZD6244 resulted in greater inhibition of Ki-67, p-RPS6, p-Erk1/2, and p-4E-BP1 compared with the monotherapy and control groups (Figure 5B).

## DISCUSSION

To date, increasingly more studies focusing on concurrent blockade of two pathways support the promise of dual-targeted strategies, e.g. targeting mTOR and MEK [20–22]. Although RCC is not much sensitive to MEK inhibitors, we hypothesize that combination with selumetinib sensitized RCC cells to everolimus treatment. Recent findings that mTOR inhibition induces compensatory MEK activation in RCC [23] and our data support this hypothesis.

This study discovered two important molecules that restrains the efficacy of everolimus in RCC. Mechanistically, RPS6 and 4E-BP1 are phosphorylated not only by mTOR, but also MEK, which limits the response of RCC to everolimus. Thus, inhibition of neither mTOR nor MEK alone was sufficient to abolish the activity of p-RPS6 and 4E-BP1. Our data also demonstrated that the p-RPS6 levels were not blocked by increasing doses of RAD001 alone. Importantly, the overall survival of patients with RCC overexpressing RPS6 and p-RPS6 tended to be shorter [24, 25], suggesting that RPS6 and p-RPS6 stimulate the pathogenesis and progression of RCC. Moreover, Pian, J. P. firstly discovered the relationship between cell cycle and RPS6 induced by the oncogene Ras [26]. We next asked whether the reduction of cell cycle proteins was associated with the blockage of RPS6 in RCC. Our data confirm that RPS6 is a key effector of inhibition of the mTOR and MEK signaling pathways in RCC.

Another intriguing finding is that RAD001/AZD6244 combination resulted in a synergistic inhibition of 4E-BP1 and p-4E-BP1. In terms of the role of 4E-BP1, several studies reported that high 4E-BP1 and p-4E-BP1 expression was associated with poor prognosis in colorectal cancer, invasive urothelial carcinoma of bladder, and non-small cell lung cancer [27–29]. Furthermore, the expression of p-4E-BP1 had a significant impact on the response of metastatic RCC patients to mTOR inhibitors; and the limited antitumor effect of mTOR inhibitors may due to the inadequate suppression of p-4E-BP1 [30]. While the RAD001/AZD6244 combination could make up this insufficiency and prevent reactivation of ERK and AKT, since 4E-BP1 inhibition is responsible for much of the activation of translation by RAS/ERK and PI3K/AKT [19].

The angiogenesis in xenograft tumor mainly depends on the environmental VEGF levels. In addition, the anti-angiogenesis effects of AZD6244 on gastric cancer were predominantly attributed to VEGF modulation [31]. Interestingly, we found that the two drugs inhibit angiogenesis as a concomitant effect by suppressing VEGF secretion from tumor cells rather than a direct effect on vascular endothelial cells. Indeed combining MEK and mTOR inhibition exert antitumor effects on CRC xenografts due to reduced expression of VEGF [32]. Taken together, selumetinib enhances the antitumor activity of everolimus against RCC by synergistically inhibiting the expression of VEGF in addition to p-RPS6 and p-4E-BP1.

As a preclinical research, further assessment on patient-derived RCC xenografts is warranted. The findings described here are only part of the complex mechanisms involved in everolimus/selumetinib combination, and other mechanisms still need to be characterized. Notably, these findings have important preclinical implications and the combination of everolimus and selumetinib may be a promising tumor-targeted therapeutic strategy for patients with renal cell carcinoma.

## MATERIALS AND METHODS

### Cell lines and inhibitors

The human RCC cell lines Caki-1, 786-O and 769-P were purchased from the American Type Culture Collection (ATCC). HUVEC cells were obtained from the cell bank of the Chinese Academy of Sciences. All cells were maintained in the appropriate medium supplemented with 10% fetal bovine serum, at 37°C in a humidified environment with 5% CO_2_. Small molecular inhibitors RAD001, AZD6244, PD0325901 and TAK-733 were obtained from Selleck (Texas, USA), and dissolved in dimethyl sulfoxide (DMSO).

### Cell proliferation and clonogenic assay

Cell proliferation experiments were performed in 96-well plates in six replicates, at a plating density of 3000 cells per well. After treatment with serially diluted inhibitors for 72 hr, cells were fixed with cold TCA (10%) at 4°C for 1 hr. Then, the plates were washed with water and stained with 0.4% sulforhodamine B (SRB) for 15 min at room temperature followed by washing with 1% acetic acid. The plates were then read on a Soft Max pro plate reader at 560 nm after staining with 10 mM Tris. Combination index (CI) was calculated using the CompuSyn software (Combo Syn, Inc., Paramus, NJ). CI < 1 indicates synergism, CI = 1 additive effect, CI > 1 antagonism.

For clonogenic assay, cells were seeded in 6-well plates at a density of 500 cells/well with the above-mentioned drugs. The drug containing medium was changed every 3 days. On day 10, the colonies were stained with crystal violet and counted.

### Cell cycle analysis

After treatment with the inhibitors for 24 or 48 hr, cells were harvested and fixed with 70% ethanol at −20°C for 24 hr. Then, cell pellets were stained with propidium iodide (Sigma-Aldrich, St. Louis, MO, USA), and incubated in the dark at room temperature for 30 min. PI fluorescence signals were assessed by flow cytometry on a FACScan flow cytometer (FACS Canto II, BD). Cell cycle distribution was analyzed using the Mod Fit software, and gated cells in G1, S or G2/M-phase were counted.

### Apoptosis

After treatment with the inhibitors for 48 hr, cells were evaluated using the Annexin V-FITC Apoptosis Detection kit (BD Pharmingen, Heidelberg, Germany) according to the manufacturer's instructions. Cells were analyzed on a FACScalibur (BD Biosciences, Heidelberg, Germany). The percentage of apoptotic cells was calculated using FlowJo software.

### Measurement of VEGF levels

1 × 10^5^ cells were seeded in 12-well plate overnight and exposed to different reagents for 24 hr. Then the expression levels of VEGF in cell culture supernatants were measured by Human VEGF Quantikine ELISA Kit (R&D Systems, USA) according to the manufacturer's instruction. The concentration of VEGF was presented as pg/ml.

### siRNA transfection

Cells were transfected with a negative control and siRNA targeting RPS6 using Lipofectamine RNAiMAX Transfection Reagent (Invitrogen, Carlsbad, CA, USA), according to the manufacturer's protocol. The sequences of the two siRNA sense strands targeting the RPS6 were: S6-110, 5′-CUUCGUACUUUCUAUGAGATT-3′; S6-453, 5′-CUAGCAGAAUCCGCAAACUTT-3′. A nonspecific oligonucleotide not complementary to any human gene was used as a negative control. All the above siRNAs were purchased from Gene Pharma (Shanghai, China).

### Western blot

20 μg total protein extracted from whole cells after lysis by RIPA buffer were separated by 8∼12% SDS-PAGE (90 min, 100 V) and subsequently transferred onto nitrocellulose membranes (60 min, 100 V). After blocked with non-fat milk for 1 hr, the membranes were incubated overnight with specific primary antibodies. Then, the membranes were incubated with secondary antibodies for 1 hr. Immunoreactive bands were detected using Amersham Hyper film ECL (GE Healthcare Life Sciences). β-actin was used as a loading control.

To explore the involved signaling pathways of RAD001 and AZD6244, the following monoclonal antibodies were used: p70S6K, p-p70S6K(Thr389), RPS6, p-RPS6(Ser235/236), 4E-BP1, p-4E-BP1(T37/46), Erk1/2, p-Erk1/2(Thr202/Tyr204) (Cell Signaling) and β-actin (Sigma).To explore cell cycle regulation proteins, the following monoclonal antibodies were applied: cyclin D1, CDK2, p-Rb(Ser807/811) (Cell Signaling) and c-Myc (Abcam). For autophagy assay, LC3II (Sigma) and p62 (Cell Signaling) were used.

### Xenograft model and treatments

Animal experiments were approved by the Animal Care and Welfare Committee of Shanghai Jiao Tong University. Five to six-week-old male nude mice (BALB/c nu/nu) purchased from Charles River (Beijing, China) were housed under pathogen-free conditions. A RCC mouse model was established by injecting subcutaneously the animals with 5 × 10^6^ Caki-1 cells in the flank region. When tumors reached approximately 100 mm^3^, the mice were randomized into four groups (*n* = 6) according to tumor volumes and body weights for the following oral treatments: vehicle control, RAD001 (2 mg/kg/day), AZD6244 (100 mg/kg/day), and a combination of RAD001 (2 mg/kg/day) and AZD6244 (100 mg/kg/day). Body weights and tumor volumes were measured twice a week using calipers. Tumor volumes were derived as V = π(length × width^2^)/6. After three weeks of treatment, the tumors were harvested, weighed, and fixed in formalin for IHC.

### Immunohistochemistry

Formalin fixed tumor tissue specimens were embedded with paraffin, cut into 5 μm sections and mounted onto slides. Then, the sections were deparaffinized, rehydrated, submitted to antigen retrieval, incubated with specific primary and secondary antibodies, and visualized under a microscope. In this study, the sections were stained with p-RPS6, p-Erk1/2, p-4E-BP1, Ki-67 and CD31 antibodies to assess signaling pathways, cell proliferation and microvessel density, respectively. For the quantification of each biomarker, 5 random images at a magnification of 200× per tumor were analyzed.

### Statistical analysis

All experiments were performed at least 3 times; data were presented as mean ± standard error of mean (SEM). To assess differences among the treatment groups, one-way ANOVA was performed followed by Dunnett post-hoc test. To assess differences in tumor sizes and therapy response over time among groups, two-way ANOVA followed by Bonferroni post-hoc test was used. *P* < 0.05 was considered statistically significant. Statistical analysis was performed using Graph-Pad Prism version 5 (GraphPad Inc., CA, USA).
